# Raman spectroscopy in head and neck cancer

**DOI:** 10.1186/1758-3284-2-26

**Published:** 2010-10-05

**Authors:** Andrew T Harris, Andrew Rennie, Haroon Waqar-Uddin, Sarah R Wheatley, Samit K Ghosh, Dominic P Martin-Hirsch, Sheila E Fisher, Alec S High, Jennifer Kirkham, Tahwinder Upile

**Affiliations:** 1 Department of Ear, Nose and Throat/Head and Neck Surgery, Calderdale and Huddersfield NHS Trust, Huddersfield UK; 2 Section of Experimental Therapeutics, Leeds Institute of Molecular Medicine, University of Leeds, Leeds, UK; 3 School of Health Studies, University of Bradford, Bradford, UK; 4 Department of Pathology Leeds Dental Institute, University of Leeds, Leeds, UK; 5 Department of Oral Biology, Leeds dental Institute, University of Leeds, Leeds, UK; 6 Department of Otolaryngology with Head & Neck Surgery, Chase farm Hospital, Enfield, U.K. and Head & Neck Unit UCLH, Euston Road, London

## Abstract

In recent years there has been much interest in the use of optical diagnostics in cancer detection. Early diagnosis of cancer affords early intervention and greatest chance of cure. Raman spectroscopy is based on the interaction of photons with the target material producing a highly detailed biochemical 'fingerprint' of the sample. It can be appreciated that such a sensitive biochemical detection system could confer diagnostic benefit in a clinical setting. Raman has been used successfully in key health areas such as cardiovascular diseases, and dental care but there is a paucity of literature on Raman spectroscopy in Head and Neck cancer. Following the introduction of health care targets for cancer, and with an ever-aging population the need for rapid cancer detection has never been greater. Raman spectroscopy could confer great patient benefit with early, rapid and accurate diagnosis. This technique is almost labour free without the need for sample preparation. It could reduce the need for whole pathological specimen examination, in theatre it could help to determine margin status, and finally peripheral blood diagnosis may be an achievable target.

## Introduction

In recent years there has been much interest in the use of optical diagnostics in cancer detection. At present a diagnosis of cancer is made on histological evaluation with possible prior cytological evidence. In some cases definitive diagnosis is only made on resection of the tumor, as prior interventions have been equivocal. Early diagnosis of cancer affords early intervention and greatest chance of cure. The ability to detect early biochemical changes associated with carcinogenesis prior to the changes a pathologist identifies would revolutionize cancer diagnostics.

Infrared spectroscopy, along with other optical techniques such as elastic scattering spectroscopy, differential path-length spectroscopy, fluorescence techniques, and optical coherence tomography have been assessed in the detection of head and neck tumors [1,2]. These have all provided encouraging results; Raman spectroscopy is in the early stages of development in relation to life sciences and this article aims to review the literature regarding its application to head and neck cancer.

## Background

The Raman Effect was first discovered by Professor Raman of Calcutta University for which he was awarded the Nobel prize in 1930 [3]. This effect is based on light's interaction with matter; as photons are directed towards target matter, most will pass through unchanged. However, some photons will come into contact with molecules in the matter. Most of these photons will interact with the molecules of the substance, exciting the particles to a partial quantum state, with the emission of a photon at the same frequency as the incident photon [4]. This process is known as elastic scattering. A smaller number of these (approximately 1 in 10^6 ^to 1 in 10^8^) photons will undergo a process called Raman or inelastic scattering. The photon is discharged from the material or 'scattered' at a differing wavelength than the incident photon and it is this wavelength shift which is recorded in Raman spectroscopy.

**Figure 1:**
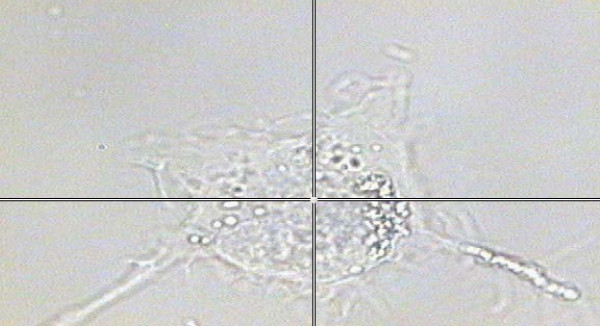
**A thyroid cell under the microscope attached to the Raman system. **This picture highlights the resolution of the Raman system, as measurements can be taken (at the cross-hairs) from a small section of a single cell (1 micro-metre squared area).

**Figure 2:**
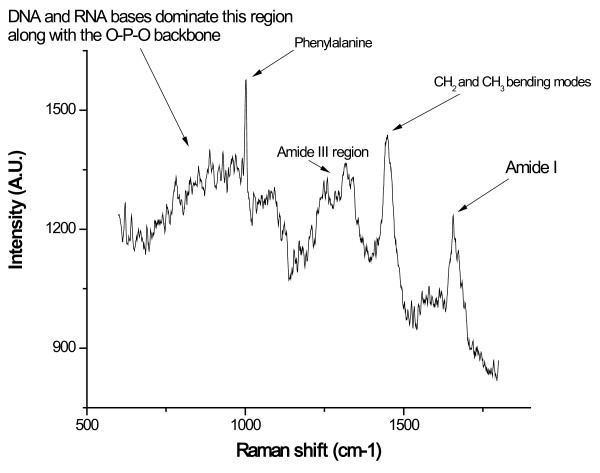
**Raman spectra taken from a single thyroid cell, depicting multiple 'spikes' attributable to biochemical species within the cell. **Some of the chemical bonds have been labeled for identification.

Raman spectroscopy is highly specific and affords the ability to take recordings from very small sections of the target sample (figure 1). The interaction of the photons with the target material produces a detailed biochemical 'fingerprint' of the sample that is characteristic for the constituent chemical bonds (figure 2). It can be appreciated that such a sensitive, high resolution biochemical detection system could confer diagnostic benefit in a clinical setting. The upper aerodigestive tract is generally more easily accessible than other regions of the body, allowing detection systems to be used in office or community settings. A Raman based diagnostic system could not only afford early tissue analysis of a solid tumour but also the possibility of detecting biochemical changes within peripheral blood of patients with cancer.

## Literature

**Figure 3:**
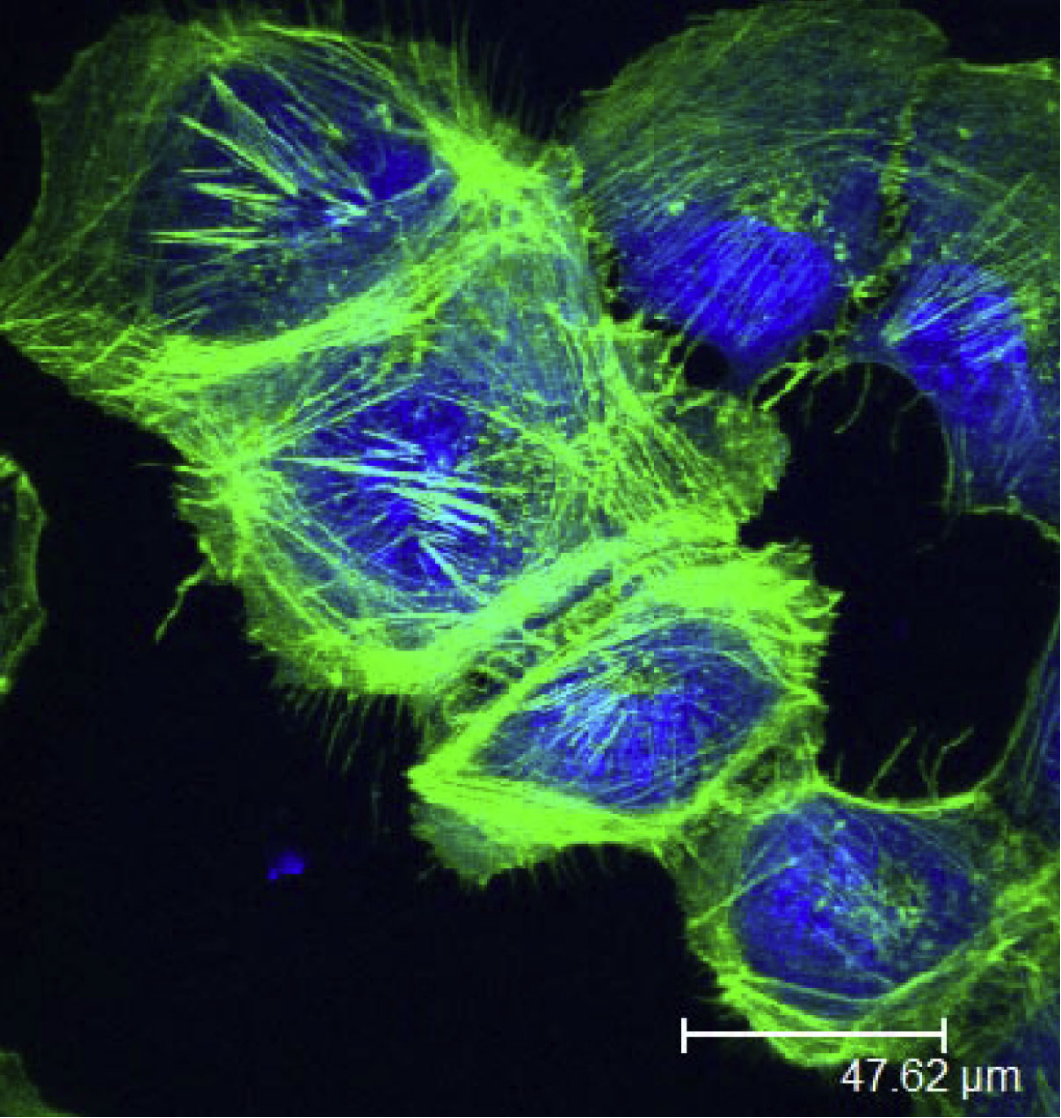
Human follicular thyroid cells viewed under confocal microscopy The cells had been stained with Alexa fluor 488 Phalloidin for actin, and To-pro3 for the nuclei.

**Figure 4:**
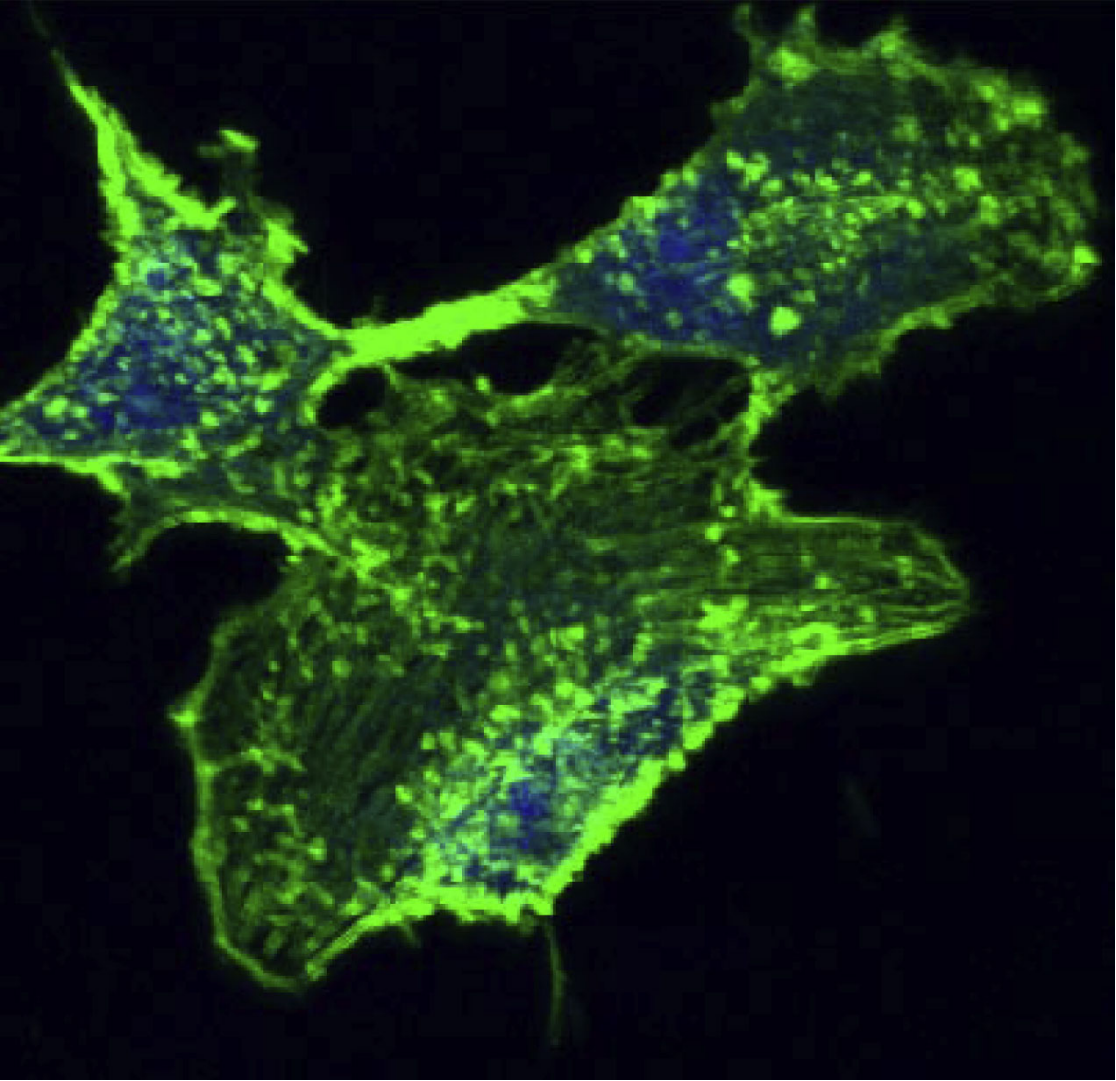
**Human anaplastic thyroid cells viewed under confocal microscopy. **The cells had been stained with Alexa fluor 488 Phalloidin for actin, and To-pro3 for the nuclei.

Harris *et al. *(2009) studied the ability of Raman spectroscopy to discriminate between two thyroid cell lines [5]. These were commercial cell lines; one of a non-cancer lineage and the other an anaplastic carcinoma cell line (figures 3 and 4). Raman spectroscopy coupled with neural network analysis gave 95% sensitivity for the anaplastic cell line and 92% for the non-cancerous cell line. Following on from these encouraging results the authors' further tested the system using five thyroid cell lines including; non-cancerous, papillary carcinoma, follicular carcinoma, medullary carcinoma and anaplastic carcinoma [6]. This time Raman spectroscopy was coupled with genetic algorithms for data analysis. Results demonstrated a relatively poor discrimination (sensitivity 61%) between cell lines of a similar genotype and ultimately phenotype, such as non-cancerous versus papillary carcinoma but when cells from opposing ends of the biological spectrum were tested such as non-cancerous versus medullary or anaplastic carcinoma, the results were much better (88% and 91% respectively). The authors are taking forward this work with testing of the algorithm using actual tissue samples.

In 2000, Stone and colleagues examined the use of Raman spectroscopy in the detection of laryngeal malignancy [7]. Fifteen *ex-vivo *biopsy specimens were obtained from patients of varying ages (18 to 79 years). They analyzed three classes of specimen (normal, dysplastic and squamous cell carcinoma). Their results demonstrated sensitivities of between 76 and 92% depending on the tissue type examined and specificities of over 90%. On further examination, the regions in the Raman spectra associated with differences between the tissue types corresponded with protein bonds and nucleic acids, leading to the discovery that nucleic acids levels are increased with malignancy. Lau *et al.*, (2005) undertook a similar study in which 47 laryngeal specimens were interrogated with Raman spectroscopy [8]. They obtained each spectrum with a five second acquisition time. The classes of specimen used were normal, papilloma and carcinoma. Sensitivities were similar to the study by Stone *et al. *(69 to 89%), with specificities ranging from 86 to 94%. The authors determined that spectral differences occurred in the DNA, amino acids, collagen and glycolipids, accounting for the ability to discriminate between the tissue types. Further experimental work on laryngeal tissue was carried out by Teh and colleagues (2008). They demonstrated that provided high quality Raman spectra could be obtained with a collection time of five seconds, discrimination between cancer and non-cancer samples provided a diagnostic sensitivity of 88% and specificity of 91%.

Lau *et al. *(2003) used Raman spectroscopy to classify tissue obtained from the post-nasal space in cancer and non-cancer patients [9]. The importance of utilizing Raman spectroscopy in the nasopharynx is the ability to detect sub-mucosal tumors associated with this cancer in this region, preventing the need for random biopsy. The study was small, observing spectra from six cancer and six non-cancer patients however, spectral differences were noted in the regions of the spectra associated with collagen, proteins and lipids.

Gniadecka and co-workers (2004) applied Raman spectroscopy coupled with neural network analysis to identify skin lesions [10]. Raman spectra were obtained from 22 melanoma samples, 41 pigmented nevi, 48 basal cell carcinoma samples, 23 seborrheic keratoses samples, and 89 normal skin samples. It was found that malignant melanoma could be discriminated from the other tissue samples based on the amide I protein region (1660 cm-1) of the spectra; sensitivity and specificity for melanoma was 85% and 89% respectively.

The main author of this article along with colleagues assessed the feasibility for Raman spectroscopy coupled with genetic algorithms to diagnose cancer through a peripheral blood sample [11]. This has the potential to revolutionize medical diagnostics with the ability for the community practitioner to have a diagnosis prior to specialist referral allowing treatment options to be booked at the earliest opportunity.

Forty patients in total were tested, twenty with an established diagnosis of head and neck carcinoma (although not all squamous cell carcinoma), and twenty aged-matched controls with respiratory ailments comprised the control group. Using a trained genetic algorithm the authors found a 75% sensitivity and 75% specificity for each cohort. When mixed samples were used to train the algorithm, the results gave the expected 50% sensitivity and specificity, providing further evidence that the algorithm was able to discriminate between cancer and non-cancer samples. The results of 75% are promising when considering what is being attempted although further testing of the algorithm with a greater number of samples and the use of different varieties of samples is also warranted.

The oral cavity is readily accessible in a clinic setting and would be ideal for the development of a Raman probe for cancer detection. Premalignant changes such as leukoplakia, erythroplakia, and erythro-leukoplakia are observed and in many cases multiple biopsies are undertaken with periods of watchful waiting. Malini and colleagues (2006) studied the ability of Raman spectroscopy to discriminate 216 pre-malignant, malignant, inflammatory and normal tissue samples from the oral cavity [12]. The discrimination between tissue types was based on surface findings. Normal healthy cell spectra originate from the lipid bi-layer, whereas inflammatory or cancerous cells contain multiple proteins on the cell surface. Disease sates were also readily distinguishable when multi-parameter tests were added in conjunction with principal component analysis.

The Raman analysis of saliva has the potential to detect malignancy within the oral cavity or orophaynx. Oral squamous cell carcinoma has a particular poor 5-year survival rate, approaching 5% [13]. This is mainly thought to be due to the late presentation of these tumours. Saliva contains many macromolecules such as proteins and nucleic acids giving a Raman signature [14]. However, Raman spectra from saliva are inherently weak, making interpretation difficult [15]. For this reason enhancement of the Raman signal using a surface-enhanced approach with gold particles is being studied [15].

## Discussion and Conclusion

As mentioned previously, Raman spectroscopy has a potential advantage over other optical methods due to its ability to provide a biochemical 'fingerprint'. However, Raman has inherently been associated with a weak signal and problems with autofluorescence. Autofluorescence is wavelength dependent and the increased use of incident photons in the near infrared region of the electromagnetic spectrum has diminished its effects. The use of lasers with a greater photon flux intensity can to some degree overcome a weak signal, also allowing spectra to be collected within a relatively short time frame in addition to overcoming the effects of fluorescence. The highly detailed molecular profile produced by Raman spectroscopy can also lead to difficulty with interpretation. Such accuracy proves difficult to determine causes of spectral differences, thus more complex data classification models are being used in an attempt to 'un-lock' it's potential. Linear discriminant analysis and principal component analysis are multivariate statistical analysis methods which are utilized along with partial least square and non-linear mapping in Raman data analysis [16-20]. However, others are utilizing more complex chemo metric classification models based around computational intelligence [21-23]. Until validated methods providing repeated accuracy on test samples are discovered, this technique will remain in the research arena.

Optical coherence tomography has recently been used *in-vivo *to determine the nature of lesions within the bladder [24]. These authors found 100% sensitivity with this optical technique for malignant lesions, also demonstrating 100% sensitivity for detecting invasion through the lamina propria. Other authors have been increasing the accuracy of OCT in cervical neoplasia demonstrating efficacy within the clinical setting [25]. In their review article, DeCoro and Wilder-Smith consider the possibilities for OCT in oral carcinoma [26]. Infrared and fluorescence spectroscopy have also been used for *ex-vivo *and *in-vivo *analysis [27-29].

At present the gold standard of cancer diagnosis is histological assessment. However, this is labour intensive; the specimen needs prior preparation; and is open to human error. Once a diagnosis of cancer has been made the patient is discussed at the multi-disciplinary team (MDT) meeting where clinical staff such as surgeons, oncologists, pathologists, and radiologists, and non-clinical staff; allied health professionals such as speech therapy and clinical nurse specialists will discuss appropriate treatment options for the patient. Patients are then seen in clinic and a definitive treatment plan agreed. As has been discussed so far early diagnosis directly affects morbidity and mortality in H&N cancer. The ability to diagnose malignant disease at the earliest opportunity allows treatment options to be planned as early as possible. If the general practitioner could provide a diagnosis of cancer to the specialist the first meeting could discuss treatment rather than further examination, booking for diagnostic biopsy and radiological investigations, and then MDT discussion, followed by treatment.

There has been a paucity of literature regarding Raman spectroscopy in head and neck cancer detection. However, others have undertaken *in-vivo *analysis of malignancies in other body regions [30-33]. Raman has success in other key health areas such as cardiovascular diseases, and dental care [34-37]. Following the introduction of health care targets for cancer, and with an ever-aging population the need for rapid cancer detection has never been greater. There is no doubt Raman spectroscopy could confer great patient benefit with early, rapid and accurate diagnosis. The authors do not believe optical diagnostics should replace pathology but aid cancer diagnosis. This technique is labour free without the need for prior sample preparation. It could reduce the need for whole pathological specimen examination, in theatre it could help to determine margin status, and finally peripheral blood diagnosis may be an achievable target.

## Competing interests

The authors declare that they have no competing interests.

## Authors' contributions

AH, AR, SW, HW-U initially drafted the manuscript. SG, DM-H, SF, AH and TU provided expert surgical and pathological advice. JK provided the biochemical expertise. All authors made an editorial contribution to the paper. All authors read and approved the final manuscript.
